# Do fast food restaurants surrounding schools affect childhood obesity?

**DOI:** 10.1016/j.ehb.2019.01.011

**Published:** 2019-02-16

**Authors:** Jebaraj Asirvatham, Michael R. Thomsen, Rodolfo M. Nayga, Anthony Goudie

**Affiliations:** a Department of Agribusiness Economics, Southern Illinois University Carbondale, IL, United States; b Department of Agricultural Economics and Agribusiness, University of Arkansas, Fayetteville, AR, United States; c Arkansas Center for Health Improvement Arkansas Center for Health Improvement (ACHI), Little Rock, AR, United States

**Keywords:** I10, I12, J13, L83, Fast food, Childhood obesity, Body mass index, School food environment, Instrumental variable estimation

## Abstract

In this study, we estimate the effect of fast food environment surrounding schools on childhood body mass index (BMI). We use two methods that arrive at a similar conclusion, but with different implications. Using school distance from the nearest federal highway to instrument for restaurant location, we find the surrounding restaurants to only marginally affect a student’s BMI measure. The effect size also decreases with increasing radial distances from school, 0.016 standard deviations at one-third of a mile and 0.0032 standard deviations at a mile radial distance. This indicates the decreasing influence of restaurants on a child’s BMI as its distance from school increases. On a subset of students who were exogenously assigned to different school food environment, we find no effect of the fast food restaurants. An important contextual aspect is that nearly all schools in this sample observed closed campus policy, which does not allow students to leave campus during lunch hours.

## 1. Introduction

Rising rates in childhood obesity have become an important worldwide health and public policy issue. Even though this has received more attention in the United States, childhood obesity is a growing problem in other countries, including those in Europe and Asia. Because the majority of children attend public schools, there is much interest in regulating the food environment in schools to promote health objectives (Story et al., 2009; Sharma et al., 2009). France, for example, banned food promotion in schools, whereas Mexico and India have banned sales of sodas and certain unhealthy foods in schools (Villanueva, 2011; Barquera et al., 2013; Khandelwal and Reddy, 2013). In the US, regulation has mostly focused on school meals and those foods stocked in school vending machines^1^. Addressing childhood obesity has become a policy priority, especially because obesity during childhood may continue into adulthood (Serdula et al., 1993).

Most public policy efforts have focused on school lunches and other food products sold within the school. There is a growing concern, however, that fast food restaurants around schools might increase caloric intake among children and thereby increase obesity (Davis and Carpenter, 2009). There are incentives for fast-food restaurants, especially national chains, to target children for increasing current sales and creating brand loyalty. This is more apparent among hamburger fast food restaurants, sandwich places, and pizzerias (Austin et al., 2005). Collectively, we will refer to these three restaurant types as *fast food restaurants*.

An important question of public policy relevance is to know whether the presence of fast food restaurants around schools have any effect on childhood obesity. We seek to identify the effect of fast food restaurants surrounding schools on a child’s BMI. Estimating the causal effect of fast food restaurants surrounding schools, however, is challenging due to: a) self-selection of families into neighborhoods; b) omitted variable bias; and c) selection of fast food restaurants in into neighborhoods according to fast-food demand. Self-selection of parents due to occupation, education or other reasons could create neighborhoods that could be similar in their attitude towards health and well-being of their children. Omitted variables in the determination of a child’s BMI could include features of the school food environment.

We address biases in the estimation using an instrumental variables approach, where the instrument is the proximity of the school to US highways that create a demand for fast food restaurants to serve highway travelers. This instrument has been used in several studies (for example, Dunn, 2010; Anderson and Matsa, 2011). In addition to this method, we estimate individual-level fixed effects for a subset of students who were exogenously reassigned to another school. This strategy follows that used by Asirvatham et al. (2018) in their analysis of peer effects. Both these methods arrive at a similar conclusion – fast food restaurants surrounding schools have a negligible effect on BMI outcomes of school children. The analytic sample is based on measured BMI records from an ongoing BMI screening program within the Arkansas public school system.

There are several ways that fast food restaurants surrounding schools could influence BMI outcomes. First, fast food restaurants and pizzerias offer calorie-dense foods that play a direct role in obesity. Even though most schools in this study observe closed campuses, a high density of restaurants around schools will generally increase the likelihood that students visit restaurants before or after school hours either unsupervised with friends or with a parent or caregiver. Second, restaurants located nearby reduces travel cost to obtain food. It could also be that proximity disincentivizes traveling longer distances to purchase healthier foods. Third, exposure to the sight of restaurants might increase the likelihood that children would choose fast foods after being exposed to fast food restaurants en route from school to home. Fast food signage could reinforce marketing messages aimed at children and increase likelihood of fast food requests on food-away-from-home meal occasions. School meals can be served as early as 10:30 am and many children will be hungry at the end of the school day. The presence of fast foods in school environment could thus increase desire for fast foods on other dining occasions regardless of whether the child consumes fast food on the way to or from school. Finally, teachers who leave campus for lunch could bring in items from restaurants (e.g., drink cups) and thereby model fast food as a reasonable meal choice. This is similar to the argument that advertisements targeting children might increase fast food consumption (Jashinsky et al., 2017). Study participants in Cambridge-shire, UK, who faced a greater exposure to fast food outlets showed higher intake of takeaway food (Burgoine et al., 2014).

## 2. Contribution to the current literature on fast food and obesity

Much of the work in this line of research provides some evidence of a positive association or correlation of obesity rates or BMI with restaurant availability or proximity (Williams et al., 2014). Most of the studies are correlational and therefore it is difficult to imply any causality. In this section, we discuss few of the studies that have conducted a more rigorous analysis going beyond correlation or association. These studies in general find a small effect.

Three studies used an instrumental variables strategy based on proximity to highways (or highway on/off ramps), to estimate the impact of fast food density on body weight outcomes among adults. In a county-level study, Dunn (2010) used the number of exits on a highway within a county to instrument for the number of restaurants in a county and finds restaurant availability to effect only females and non-whites in medium-density counties in 11 states. Such a relationship was not found in rural or low-density counties. In a similar study on residents of central Texas, Dunn et al. (2012) reported only non-whites to exhibit higher obesity rates in response to fast food exposure. These authors also used distance to the nearest major highway as an instrument for the fast-food restaurant. Anderson and Matsa (2011) find no effect of fast food restaurants. They also exploit the variation in travel costs of local residents to restaurants along interstate highways, which primarily serves travelers. Using related data, they observed that obese individuals complement eating outside by consuming nutritionally deficient or “junk foods.” They conclude that policies focusing solely on regulating fast food restaurants may not achieve any significant reduction in BMI.

The above studies examine adult populations. Two studies analyze fast food restaurants around schools and find fast food restaurants to significantly affect childhood obesity rates. Currie et al. (2010) uses a cross-sectional sample of students from grade 9 in public schools in California. In their study, the effect is identified by comparing groups of individuals at only slightly different distances to a restaurant (i.e., of only one-tenth of a mile). They measure changes in exposure, with obesity being measured as the fraction of obese students at the grade-level in a school. In their study, they use changes in obesity prevalence in the same grade (grade 9) but across different years. Alviola et al. (2014) study the public school sample in Arkansas. Following the earlier studies on adult populations, their instrumental variable was the distance of school from the nearest highway. Using school-level cross-sectional data, they report coefficients that measure the difference in obesity rates across schools that face different restaurant counts. Their study estimates a 1.23 percentage point increase in school obesity rates in response to an additional restaurant within a one-mile radius from a school. In this article, we also focus on Arkansas public schoolchildren. However, unlike Alviola et al.’s (2014) school-level analysis, this study uses individual-level analysis of BMI z-scores over time; i.e, we use *student*-level panel data in contrast to the *school*-level cross-sectional data they used. In addition to an identification strategy based on an IV estimation using distance of schools to the nearest highway as an instrument, we also employ an additional identification strategy based on a plausibly exogenous reorganization of public schools that caused some children to be reassigned to a new school zone. Hence, these re-assigned students face a new food environment in their newly assigned school.

The present article extends these studies in several ways. One, we have a unique panel dataset, which gives us the ability to measure changes within a student and estimate fixed effects at the individual level. Several of the previous studies use obesity rates (proportion of obese adults or children) or obesity status (a binary variable). We are able to estimate the impact on precise changes in BMI in terms of standard deviations of the child’s BMI z-score. Two, in contrast to several past studies, the BMI data used here are measured by trained personnel, as opposed to being self-reported. Three, we control for the commercial food environment near a child’s residence with precise geographic information. It is important to control for other sources of calories because this could influence the quality and quantity of food consumed. Four, asmentioned above, this study uses two methods to identify the effect of fast food availability around schools on children’s BMI z-scores: IV and the exogenous assignment of students to nearby schools as a result of a court-mandated restructuring program. These are discussed in detail in the section Identification Strategies.

## 3. Methods

The primary aim of this study is to estimate the effect of fast food restaurants surrounding schools on childhood obesity. The variation in a student’s exposure to fast food restaurants comes in two ways. One, change in the count of restaurants surrounding schools. Two, students moving to different schools either because of a natural progression through the public school system or by just relocating within the state and, thereby, to another school. In this dataset, about 42 percent of the students relocated but remained in the public schools in Arkansas. A child’s BMI could be influenced by several environmental factors inside schools, outside of schools, within the family, and within the community, some of which could be related to the density of fast food restaurants. Our data allow us to control for several observable factors. However, note that the coefficient on the number of fast food restaurants takes into account only the covariation between the restaurant count around the school and BMI z-score. Food intake from home might bias the coefficient of interest, but we include variables that capture the commercial food environment around a student’s residence, including distance to nearest grocery, dollar store, convenience store, fast food, pizzeria and sandwich place. To the extent household behaviors towards food consumption persist with time, the inclusion of student-level fixed effects can be advantageous as they difference out unobserved time-invariant factors that might be correlated with the variable of interest.

In terms of the features of the food environment within schools, all schools were subject to Act 1220 of 2003. Act 1220 required nutrition standards to be applied to all foods and beverages sold or made available to maintain a healthy school environment. For example, Act 1220 requires 50% vended beverages to be a healthy choice such as water, 100% fruits juice and low-fat/fat-free milk. Act 1220 was in effect across Arkansas during the entire study period. Thus, we do not expect wide variation in within school environment across schools in terms of the food and physical education/activity environment. Other factors within schools could play some role. For example, peers may influence diet and physical activity choices that might then influence body weight. One challenge with including peers’ variable is that it brings with it into the model a host of other biases. As described in Asirvatham et al. (2018), these biases could further complicate the model. We, therefore, do not include peers’ weight variable but include school-level percentages of different race groups and meal status. Both of these are correlated with obesity and, therefore, to some extent account for peers influence (Boyd et al., 2011). Nevertheless, we acknowledge that the methods used here can only partially addresses the biases created by unobserved changes within the school environment during the study period.

There could also be common factors at the community-level that might drive restaurant counts. Consider a town with a population that does not demand high calorie foods. Such a population might also have lower restaurant counts in the near vicinity of schools. One implication of such demand is that the lower income population might demand lower-cost food products, which are often provided by fast food restaurants. To address all of these biases, we use an instrument that drives the fast food restaurants’ location choice.

## 4. Identification strategies

We estimate the effect on two samples, one on the whole student population and the other on a subsample of students who were exogenously reassigned to other schools in response to a court-mandated reorganization of schools and districts. This allows us to test for possible mechanisms of the effect and propose some recommendation to reduce its effect on BMI outcomes. Although complementary, the analyses we conducted using these two samples provide different insights. We utilized the instrumental variables approach on the larger student population for whom all information was available, and then exploited a natural experiment of exogenous school assignment of a subset of students in response to a court-mandated school restructuring. Below we discuss both methods in detail.

### 4.1. Instrumental variable (IV) method

The instrument we use is the distance of the school to the nearest interstate or US highway. This has been well established in the literature. Dunn (2010); Anderson and Matsa (2011), Dunn et al. (2012), and Alviola et al. (2014) use some measure of highway proximity as an instrumental variable for fast food restaurants. Fast food restaurants locate in places where there is demand for the products they offer. By locating closer to highways, fast food restaurants are able to profit from the demand from travelers, but this could exogenously increases exposure to fast-food restaurants for those living near major highways, or in our case for schools located close to major highways. The identifying assumption is that nearness to highway is a factor in restaurant location decisions. That is, nearness to highway is a factor in restaurant location decisions because proximity to interstate and US highways might bring in firms supplying services and products to travelers.

Using County Business Patterns data, Dunn (2010) shows that interstate exits drive the location of fast food establishments in way that is different from other businesses. He also shows that number of interstate exits is not correlated with other healthy behaviors, such as fruit and vegetable consumption and physical activities. Dunn (2010) discusses both aspects of demand and supply that could bias an estimate. For example, higher demand for fast food could lead to more restaurants. On the other hand, residents with preference for health could enforce restrictions on location and number of fast food restaurants. He concludes that the direction of the bias is generally positive.

In our context, schools factoring in distance from state and federal highways might violate the exclusion restriction. This violation would occur because a school’s decision and a restaurant’s decision are influenced by the same explanatory variable. Even though some states do stipulate school construction to be at a distance from highways, Arkansas only stipulates distance from any source of sound that produces 65 decibels sustained and 75 decibels peak^2^. Thus, we argue that the distance of schools from highway in the context of policies in place in Arkansas does not violate the exclusion restriction.

During our study period, the locations of highways are fixed. However, students change schools as part of a natural progression through the public-school system and this creates time series variation in the distance between the child’s school and highway. The instrument used in the literature, in general, considers only interstate highway, but due to the sparseness of interstate highways in Arkansas, as shown by Alviola et al. (2014), we consider both interstate and US highways for our instrument.^5^ The standard way to include the IV in an equation is to use it as a single variable. In this study, we include it in two different ways. Firstly, as a single continuous variable. Secondly, to control for a non-linear relationship, the IV is split into segments based on different radial distances from the school to the highway. Since we estimate the effect at one-third, two-thirds, and a mile, the instrument is split into four parts, namely one-third mile, two-thirds mile, a mile, and more than a mile. A school that is a third of a mile away from the nearest federal highway will have the specific distance in the subvariable for one-third mile, with zeros in other sub-variables. This is tantamount to relaxing the assumption that the instrument has a unique linear relationship with the endogenous variable.

### 4.2. Exogenous assignment to schools

In addition to the instrumental variable method, we analyze a subsample of students who were exogenously assigned to different schools. This subsample of 2739 observations constitutes a small fraction of the total 1,352,696 observations in the main analysis sample, but provides a complementary analysis to the IV methods explained above.

The strategy is similar to that pursued by Asirvatham et al. (2018) in their analysis of peer effects using the Arkansas BMI data. As explained in this earlier study, the exogenous reassignment was created by a court mandated restructuring of public schools. In 1992, the Lake View School District and other plaintiffs claimed that the school funding system was unconstitutional. In the *Lake View School District No. 25 v. Huckabee* case, the Arkansas Supreme Court ruled that the state educational funding was unconstitutional. To meet the court mandate, the State passed the Public Education Reorganization Act, Act 60, during the Second Extraordinary Session of 2003.

School reorganization thus occurred to overhaul the public school funding system. Since the primary motivation was not to restructure the schools to improve students’ health and because the legislation was passed in a special session of the state legislature in response to the court ruling, we posit that this restructuring created an exogenous school assignment for the students it affected.

There were two ways the State sought to comply with the court decision: either through consolidation or annexation for all districts with fewer than 350 students. Consolidation involved cutting administrative overhead by bringing schools and/or school districts under fewer management personnel. Annexation required physically closing school locations and have the children attend a nearby school that met the criterion set by the legislature. Thus, annexation created a change in school of attendance that was plausibly exogenous.

An exogenous reassignment to other schools more directly addresses the issue of self-selection into schools. This reassignment, we argue, is largely uncorrelated with observables and unobservables that determine BMI outcomes. This is especially valid because the students are not in their original school attendance zone, which then largely rules out common factors or linkages between student BMI outcomes and restaurant location around their re-assigned school. In the results section, we discuss differences between annexed schools and reassigned schools.

## 5. Data

In an effort to combat high rates of childhood obesity, the Arkansas General Assembly passed the Act 1220 of 2003. Among other things, this legislation mandated that public schoolchildren be assessed for BMI beginning in the 2003–2004 school year. BMI screenings have been ongoing since that time. We use these BMI screenings as the main data source in this study. The Arkansas Center for Health Improvement (ACHI) led the development and implementation of the state-wide BMI assessment process.^13^ ACHI developed a statewide protocol for standardized measurements across the state. Height and weight measurements are measured by trained personnel in schools and are reported to ACHI. The dataset we use includes BMI z-scores, race, gender and participation in free or reduced lunches. These data also include the weight status of each child, which based on reference growth charts from the Centers for Disease Control and Prevention (CDC). Only those students with at least two BMI observations are included in our analysis sample. Medical professionals and the Centers for Disease Control & Prevention (CDC) use BMI z-scores as a general indicator to track body weight progress among children. As a child grows, so does his/her body weight and height. The BMI z-score takes into account this growth and development, which also differs by sex. The BMI z-score provides a uniform measure of body weight across our sample that contains children that differ by age and sex.

Our empirics are based on a panel dataset covering the years 2004–2010. One problem we confronted in assembling the data set is that state policy relating to the frequency of BMI measurement changed during our study period. From 2004 to 2007, the BMI of school children was measured annually for all grades. Thereafter, BMI was measured and reported only for children in even-numbered grades inclusive of kindergarten. Thus, we have BMI prevalence rates for students from all grades from 2004 through 2007, but only for students in even grades after 2007. The non-reporting of obesity prevalence in odd grades after 2007 should not bias our estimates, since the decision to stop measuring the BMI of children in odd-numbered grades was exogenous in that it was not made by the child, the child’s family, or the child’s school. However, this change in reporting does affect our ability to take into account BMI changes in a consistent fashion over time.

Another source of data we bring into the analysis is location of food businesses. These are based on Dun & Bradstreet business lists and include restaurants, grocers, and other food stores. Details on the construction of the food environment are provided in the Appendix. Using Geographic Information System (GIS) software, we create measures of the commercial food environment around schools and residences. These variables measure the number and type of restaurants at varying radial distances from schools in the increments of a third of a mile up to a mile. Specifically, we measure the number of fast food restaurants, sandwich places and pizzerias. Measures of the food environment around a student’s residence include distance to the nearest grocery store, dollar store, convenience store, fast food restaurant, pizzeria, and sandwich place.

Fast food restaurant counts may reflect local demand for fast food. To the best of the authors’ knowledge, all but one school had a “closed campus” policy during the study period. Seniors in this high school had the option of eating out during the lunch period with school permission. Students in the sample would not generally have sanctioned access to fast foods during the school day suggesting that access would be primarily before and after school hours. We also restricted the sample to those less than 18 years old.

### 5.1. Annexed sample

Table 1 presents the summary statistics of the entire population of students included in the analysis (called the general sample) and also compares it to the students who were reassigned to a different school (called the annexed sample). Students in the general sample differ from those students who were reassigned to a different school in certain characteristics. The BMI z-scores in the annexed sample are 0.05 standard deviations larger than the overall sample. There is a negligible difference across gender proportion. Annexed schools, however, had 27 percentage points more African American students and 21 percentage points less Caucasian students. The annexed sample also had a higher percentage of students qualifying for free lunches, 65% compared to 43% among all schools. In terms of the commercial food environment, students in annexed schools were further away from all of the measured food establishments. This sample also had slightly more rural students. The summary statistics generally reflect the communities that were affected by this school restructuring process. For instance, this reform closed smaller schools and integrated them into larger schools. Smaller schools are predominantly in the rural areas. Overall, differences in school-level demographic characteristics between the general sample and the annexed schools are markedly different in terms of race, household income, urbanization and access to some of the most important food establishments.

One concern in the restructuring process is if students were reassigned to schools with similar characteristics or in similar neighborhoods. Asirvatham et al. (2018) assessed differences in the schools that closed and the schools to which students from the closed schools were reassigned. Data on children from the sending schools, i.e., annexed schools, constitute the second analytic sample we used in our analysis. This exogenous assignment addresses the issue of self-selection because, had it not been for the annexation, these students would not otherwise be attending a school outside of their original attendance zone. Asirvatham et al. (2018) found significant differences in percent free and reduced lunch between sending schools and receiving schools indicating that the schools differed in socioeconomic characteristics. However, by the nature of the school restructuring process and difference in student population characteristics, we can affirm that a student’s current BMI is unlikely to be correlated with unobserved factors at the new school.

## 6. Econometric model

Our basic model to estimate the effect of fast food restaurants surrounding schools on student BMI is:

(1) $${\text{Y}}_{\text{it}}=\beta_{0}+\beta_{1}{\text{R}}_{\text{k}\left(\text{i}\right)\text{t}}+\beta_{2}{\text{X}}_{\text{it}}+\beta_{3}{\text{X}}_{\text{k}\left(\text{i}\right)\text{t}}+\beta_{4}{\text{F}}_{\text{it}}+{\text{U}}_{\text{ikt}},$$

where *Y*_*it*_ is the BMI z-score of the *i*^th^ student in time *t*; *R*_*k(i)t*_ is the restaurant counts around the *k*^th^ school of the *i*^th^ student at time *t*; *X*_*it*_ is a vector of student *i*’s characteristics; *X*_*k(i)t*_ is a vector depicting demographic student characteristics at the school-level in school *k* where student *i* attends, including proportion of students of different race categories and gender and percent qualifying for free and reduced lunch; *F*_*it*_ is the vector of commercial food environment near the residence of student *i* which includes distance to nearest grocery, dollar store, convenience store, fast food, pizzeria and sandwich place; and *U*_*i*_ is the error term which equals μ_i_ + ε_it_, where *μ*_*i*_ is the unobserved time invariant component and *ε*_*it*_ is the spherical error term. Variables measuring different aspects of the commercial food environment around student’s school and residence were constructed using GIS software and geolocation of the schools, food establishments and student residence. To tease out the effect of distance of the fast food restaurants from schools, we developed three variables measuring the number of fast food restaurants within different radial distances from school, namely one-third of a mile, two-thirds of a mile, and a mile. Besides the number of restaurants at different radial distances, we also created two sets of variables with different combinations of restaurants. One included only fast food restaurants and the other included fast food, sandwiches and pizzerias.

The panel nature of the data and the amount of information on students, schools and food environment allow us to control for individual and school-level characteristics, and also to use student-level fixed effects that further reduces the endogeneity bias due to omitted variables in the estimate. Since there could be year-to-year changes in a student’s BMI that if not accounted for might bias the estimates, we also estimate a two-way FE model by adding binary variables for different years in the data period. Thus, the two-way FE models include both student-level fixed effects and year fixed effects. Note that the observations are annual so year fixed effects would capture time-invariant year to year changes in the BMI that are not captured by the included right-hand side variables.

## 7. Results and discussion

As discussed above, our research objective is to estimate the impact of fast food restaurants surrounding the schools on a child’s BMI z-score, and check if there are any differences in the effects by radial distance of restaurants from school. In this section, we first present results from the pooled OLS (top panel, Table 2) and student fixed effects models (bottom panel, Table 2). The pooled OLS shows that the effect, though likely biased, is very small or zero in magnitude and that the sign of the coefficient is always negative, which is inconsistent with our prior expectation that fast food exposure should be positively related to BMI.

Importantly, healthier parents choosing healthier neighbor-hoods might create positive bias. On the supply side, restaurants locating in neighborhoods with a larger proportion of lower income families might at least show some restaurant effect. Pooled OLS, however, assumes all observations are independent. Our data have multiple observations on each student and therefore we are able to obtain *within estimates* by controlling for the student-level fixed effects. These results are in the bottom panel of Table 2. Some of the estimates are positive in sign but are very small in magnitude. Moreover, results are robust to inclusion of grade effects or additional controls for food stores around children’s residences. Controlling for the commercial food environment surrounding the residence of each student had little impact on the estimates once student-level time-invariant unobserved factors are accounted for.

Even though we control for unobserved time-invariant factors at the student-level beyond the residential food environment, we cannot entirely rule out the self-selection bias. This could potentially bias the estimate because parents choose where they live. For example, an occupation requiring frequent long-distance travel might increase the likelihood of choosing a residence near highways.

As previously discussed, our first identification strategy is to instrument for the restaurant counts. Results from fixed effects IV method are presented in Tables 3 and 4. The heteroskedasticity-robust first stage regression results in Table 3 indicate that average distance of schools from federal highways (the instrument) is a very significant and strong predictor of restaurant counts surrounding schools. The negative coefficient indicates that the number of restaurants decrease as the distance of the school from the highway increases. Large F-statistics are indicative of high correlation between restaurants counts and the instrument.

On the smallness of the magnitude, it should be noted that more than 50% of the schools did not have restaurants within the certain defined radii at some point during the study period. The sign suggests that the farther the average distance of a school from nearest highway, the lesser is the number of restaurants locating near schools. The reduced form fixed effects regression also yielded significant estimates of the instrument. Despite the absence of fast food restaurants for a large number of schools, the magnitude at one-third radial distance suggests a 2.1 percent increase in BMI z-score, 0.0147 standard deviations from the mean 0.704 SD. At a two-thirds mile radial distance, the estimate decreased by a third and at a mile radius, the estimate further decreases by a fifth.

The instrumental variables estimates show interesting results. First, the general result is that restaurants around any defined radii have at least some effect on student BMI. Second, within the same group of restaurants, the effect decreases as the radial distance increases. This reflects the fact that as a restaurant locates further away from a school, its influence on BMI begins to wane. This could indicate the reduced effect on calorie intake as distance from a school increases or that distance from school does matter – even though the effect is small. Thirdly, among different groups in general, the estimate is higher when the fast food measure excludes sandwich places and pizzerias. At a third of a mile radius, fast food restaurants alone show an increase of 0.024 standard deviations in BMI z-score compared to 0.015. This seems counterintuitive, but keep in mind that only about 50% of the schools had fast food restaurants. This relatively smaller magnitude could indicate that the effect of sandwich places and pizzerias might be much lower than the fast foods itself. Thus, the IV method finds a very modest effect of the restaurants surrounding schools on a student’s BMI.

Our complementary analysis of the annexed sample is consistent with the findings above in that there is no evidence of a large fast-food effect on BMI (Table 5). It is much smaller. Both OLS and fixed effects estimates show no significant effect at any radial distance from the school. The observations on reassigned students constituted less than a quarter percent of the total observations used in the analysis above. The regression model used to the obtain the results in Table 3 include similar control variables to thethose usedinthepanel IV models reported above. As discussed above, this sample is different from the general sample analyzedin several respects. Those differences could partly explain why no effect is observed in the annexed sample compared to only a marginal effect in the general sample. Compared to the general sample students in the annexed sample had to travel further away because the school in their attendance zone closed which may have prevented access to restaurants before and after school-hours. The OLS and the fixed effects estimates show high standard errors.

The results presented so far analyze all students and include several covariates that might influence a student’s BMI. In the Appendix, we ran additional regressions to examine if the restaurant effect might vary by age or by geographic areas based on city centers or population densities. Specifically, specifications based on age group present estimates for elementary, middle, and high schools (Tables A1–A3, respectively). Estimates from regressions for rural and urban schools are presented in Tables A4 and A5, respectively. The results show some differences by age but nothing of economic significance. The main conclusions of the paper appear to hold across the different age categories. We also do not find any difference across rural and urban areas.

## 8. Conclusions

In this study, we estimate the effect of the number of fast food restaurants surrounding schools on the body mass index (BMI) z-score of school children. The study uses individual-level panel data from students attending public schools in Arkansas. The results from the two methods on two samples drawn from the same student population are similar. First, an instrumental variable strategy is employed using an instrument that has been validated by previous studies. A school’s distance from the nearest highway is used to instrument for the endogenous variable of interest, the restaurant count surrounding the school at specified radial distances. The identifying assumption is that fast food restaurants located by the highways cater to the highway travelers. We use the same instrument in two ways for efficient estimation. In the first specification, we use a single variable. In the other specification, we separate the instrumental variable into parts based on its distance from the highways.

The same instrument is used in two different specifications, and both arrive at similar results. Second, we exploit a natural experiment that resulted when a number of Arkansas schools were reorganized in response to a state Supreme Court decision on school funding. This created an exogenous reassignment of students to schools other than the one in their attendance zone. Students in this exogenously assigned sample have different exposure to restaurants surrounding schools. In fact, given their distance and time to travel to a school that is located further away, it is unlikely that they would have had the opportunity to visit a fast food restaurant while taking the school bus. Thus, the two estimates, although complementary, have different implications.

This study finds the effect of restaurants surrounding schools on childhood obesity to be negligible. Our point estimate, while very small, is highly significant. The smaller estimate might cultivate a laissez-faire attitude towards regulating restaurants around schools, but the findings reported here must be interpreted within the context of the study. Most Arkansas schools to had closed campus policies during the study period that meant children would only have access to outside restaurant food before or after school hours. Hence, replicating our study in other contexts to test the robustness of our findings would be warranted.

## Figures and Tables

**Table 1:** Summary statistics.

Variable	Full Analytic Sample (N = 1,362,306)	Annexation Sample^a^ (N = 2739)
BMI z-score	0.704	0.754
***Race*** (percent)^b^		
Caucasian	66.45	45.3
African American	21.94	48.9
Hispanic	6.73	3.4
Native American	0.37	0.2
Asian	1.24	0.2
***School meal*** (percent)		
Free meals	42.85	65.2
Reduced meals	9.53	10.7
***Nearest food store*** (miles)		
Nearest grocery	3.09	4.7
Nearest dollar	2.92	4.9
Near convenient store	1.61	2.9
Nearest fast-food	2.60	4.2
Nearest pizzeria	4.20	6.4
Nearest sandwich	2.80	5.1
***Other demographics***		
Female (percent)	48.6	48.8
Age (months)	134.8	125.3
Rural (percent)	30.0	40.2
Urban (percent)	57.1	45.6

a This population includes all students in grades 1–9 and who have at least two annual observation post annexation.

b Race other than those listed above is not included in the Table.

**Table 2:** Estimates of density of fastfood restaurants (FF), sandwich places (SW) and pizzerias (PZ) surrounding schools on student’s BMI z-score.

Model	Variable (miles)	Base Model (BM)	BM + grade FE	BM + grade FE + Food Environment
OLS	FF, SW & PZ (1/3)	−0.0011658 (0.00095281)	−0.00069997 (0.00095475)	0.0011658 (0.0009574)
	FF, SW & PZ (2/3)	−0.00236391*** (0.00042873)	−0.00246344*** (0.00042967)	−0.0009603** (0.0004356)
	FF, SW & PZ (1)	−0.00201689*** (0.00027862)	−0.002020111*** (0.00027929)	−0.0007813*** (0.0002866)
	FF (1/3)	0.00092471 (0.00138714)	−0.0021661 (0.00139228)	0.0018418 (0.0013942)
	FF (2/3)	−0.00384492*** (0.00071439)	−0.0039002345*** (0.0007154)	−0.0018201*** (0.0007233)
	FF (1)	−0.00387424*** (0.00048186)	−0.00394801*** (0.00048323)	−0.0021296*** (0.0004926)
Fixed Effects	FF, SW & PZ (1/3)	0.0007181 (0.00049631)	0.00061789 (0.0004971)	0.0005299 (0.0004974)
	FF, SW & PZ (2/3)	0.00037238 (0.00023205)	0.00037723 (0.00023251)	0.0002952 (0.0002332)
	FF, SW & PZ (1)	0.00027201* (0.0001574)	0.00028487* (0.00015776)	0.0002097 (0.0001585)
	FF (1/3)	0.00089283 (0.00070364)	0.00047178 (0.00070548)	0.0003469 (0.0007059)
	FF (2/3)	0.00002974 (0.0003881)	0.00005405 (0.00038875)	−0.0000816 (0.0003895)
	FF (1)	0.00010497 (0.00026805)	0.00005186 (0.00026894)	−0.000073 (0.0002699)

*Note:* Regressors in the base model (BM) regression model include student age, race, gender, rural/urban area of residence, participation in school free/reduced lunch program, and year dummy variables. BM + grade FE has all the variables in BM plus the grade fixed effects. The last column reports estimates from a model that has all variables as in BM, grade fixed effects and the commercial food environment around student residence. OLS estimates and student fixed effects (FE) estimates differ only in the fact that the FE models include student fixed effects in addition to the other regressors listed. School-level clustered standard errors are in parentheses. N = 1,362,696.

Robust clustered standard errors are given in parenthesis.

*** Significant at the 1 percent level.

** Significant at the 5 percent level.

* Significant at the 10 percent level.

**Table 3:** Fixed effects iv estimates of density of fastfood restaurants (FF), sandwich places (SW) and pizzerias (PZ) surrounding schools on student’s BMI z-score.

Variable (miles)	IV estimates	First Stage	Reduced Form	F-stat
FF, SW & PZ (1/3)	0.0146856*** (0.0043494)	−0.0000617*** (0.000000806)	−0.000000907*** (0.000000268)	5872
FF, SW & PZ (2/3)	0.004594*** (0.0013601)	−0.0001974*** (0.00000219)	−0.000000907*** (0.000000268)	8103
FF, SW & PZ (1)	0.0029035*** (0.0008596)	−0.0003123*** (0.00000319)	−0.000000974*** (0.000000268)	9610
FF (1/3)	0.0244387*** (0.0072401)	−0.0000371*** (0.000000532)	0.000000974*** (0.000000268)	4856
FF (2/3)	0.0075479*** (0.002235)	−0.0001201*** (0.00000127)	−0.000000974*** (0.000000268)	8949
FF (1)	0.0045204*** (0.0013385)	−0.0002006*** (0.00000189)	−0.000000974*** (0.000000268)	11,225

*Note:* Regressors in each regression model include student age, race, gender, rural/urban area of residence, participation in school free/reduced lunch program, year dummy variables, the commercial food environment around student residence and student fixed effects. School-level clustered standard errors are in parentheses. US and interstate highways are used as instruments. N = 1,352,696.

** Significant at the 5 percent level.

* Significant at the 10 percent level.

*** Significant at the 1 percent level.

**Table 4:** Fixed effects split-iv (continuous) estimates of density of fastfood restaurants (ff), sandwich places (SW) and pizzerias (PZ) surrounding schools on student’s BMI z-score.

Variable (miles)	IV estimate	IV part^a^ (mile)	First Stage (estimate)	Reduced Forms (estimate)
FF, SW & PZ (1/3)	0.0021417 (0.0014272)	0 to 1/3^rd^	−0.001246*** (0.0000267)	−0.00000427 (0.00000716)
		1/3rd to 2/3^rd^	−0.0016149*** (0.0000132)	−0.00000642** (0.00000337)
		2/3rd to 1	−0.0008391*** (0.00000948)	−0.000000186 (0.000000227)
FF, SW & PZ (2/3)	−0.002606*** (0.0007005)	0 to 1/3^rd^	−0.0010658*** (0.0000584)	−0.00000427 (0.00000716)
		1/3rd to 2/3^rd^	−0.0018108*** (0.0000275)	−0.00000642***,* (0.00000337)
		2/3rd to 1	−0.0016538*** (0.0,000,186)	−0.000000186 (0.000000227)
FF, SW & PZ (1)	−0.0010169** (0.0005787)	0 to 1/3^rd^	0.0001297 (0.0000814)	−0.00000427 (0.00000716)
		1/3rd to 2/3^rd^	−0.0009549*** (0.0000401)	−0.00000642** (0.00000337)
		2/3rd to 1	−0.0010386*** (0.0,000,268)	−0.000000186 (0.000000227)

*Note:* Regressors in each regression model include student age, race, gender, rural/urban area of residence, participation in school free/reduced lunch program, year dummy variables, the commercial food environment around student residence and student fixed effects. School-level clustered standard errors are in parentheses. US and interstate highways are used as instruments. N = 1,352,696.

a First stage results for the IV part greater than 1 mile are not presented here. In every case is negative and significant at 0.01.

*** Significant at the 1 percent level.

** Significant at the 5 percent level.

* Significant at the 10 percent level.

**Table 5:** Estimates of density of fastfood restaurants (FF), sandwich places (SW) and pizzerias (PZ) surrounding schools on student’s BMI z-score for the annexation sample.

Model	Variable (miles)	Estimate
OLS	FF, SW & PZ (1/3)	0.0256623 (0.058483)
	FF, SW & PZ (2/3)	−0.0158216 (0.0298973)
	FF, SW & PZ (1)	0.0049307 (0.0262291)
	FF (1/3)	−0.0762247 (0.072834)
	FF (2/3)	0.0300403 (0.055523)
	FF (1)	−0.0175953 (0.0388795)
Fixed Effects	FF, SW & PZ (1/3)	−0.0027338 (0.0172865)
	FF, SW & PZ (2/3)	−0.0143413 (0.0096316)
	FF, SW & PZ (1)	−0.0112431 (0.0089123)
	FF (1/3)	0.0145423 (0.0241696)
	FF (2/3)	−0.0069048 (0.0141348)
	FF (1)	−0.006933 (0.012964)

*Note:* Regressors include student age, race, gender, rural/urban area of residence, participation in school free/reduced lunch program and year and grade dummies. Base model also includes school fixed effects. The student fixed effects (FE) model includes student fixed effects in addition to the other regressors listed. School-level clustered standard errors are in parentheses. N = 2739.

*** Significant at the 1 percent level.

** Significant at the 5 percent level.

* Significant at the 10 percent level.

Robust clustered standard errors are given in parenthesis.

## Footnotes

1 ‘Healthy, Hunger-Free Kids Act of 2010’ [42 USC 1751].

2 On transportation routes, Arkansas requires noise from air and motor vehicle traffic to not exceed sound decibel level of 65db sustained and 75db peak – Arkansas Rule and Regulations Governing the Minimum Schoolhouse Construction Standards; Arkansas Code 6–20-1406.

## Appendix A.

**Table A1:** Estimates of density of fastfood restaurants (FF), sandwich places (SW) and pizzerias (PZ) surrounding schools on student’s BMI z-score among **ELEMENTARY** students.

Variable (miles)	Base Model (BM)	BM + grade FE	BM + grade FE + Food Environment
FF, SW & PZ (1/3)	0.0014376 (0.002094)	0.0014488 (0.0020926)	0.0025444 (0.0020956)
FF, SW & PZ (2/3)	−0.001168 (0.0008928)	−0.0010017 (0.0008922)	−0.0000156 (0. 0,008,994)
FF, SW & PZ (1)	−0.0008875 (0.0005655)	−0.0007106 (0.0005655)	0.0000721 (0.0005736)
FF (1/3)	0.0028932 (0.0031002)	0.0028674 (0.0030987)	0.0038325 (0.0031031)
FF (2/3)	−0.0022872 (0.0014039)	−0.0019864 (0.001403)	−0.000749 (0.001412)
FF (1)	−0.0026321*** (0.0009547)	−0.0023792** (0.0009543)	−0.001328 (0.0009646)

*Note:* Regressors in the base model (BM) regression model include student age, race, gender, rural/urban area of residence, participation in school free/reduced lunch program, year dummy variables, and lag of the included restaurant count in the model. BM + grade FE has all the variables in BM plus the grade fixed effects. The last column reports estimates from a model that has all variables as in BM, grade fixed effects and the commercial food environment around student residence. School-level clustered standard errors are in parentheses. N = 370,123.

* Significant at the 10 percent level.

*** Significant at the 1 percent level.

** Significant at the 5 percent level.

**Table A2:** Estimates of density of fastfood restaurants (FF), sandwich places (SW) and pizzerias (PZ) surrounding schools on student’s BMI z-score among **MIDDLE** school students.

Variable (miles)	Base Model (BM)	BM + grade FE	BM + grade FE + Food Environment
FF, SW & PZ (1/3)	−0.0030406 (0.0030211)	0.0035474 (0.00302)	−0.0034874 (0.0030293)
FF, SW & PZ (2/3)	0.0025396* (0.0014609)	0.0015756 (0.0014658)	0.0022654 (0.0014757)
FF, SW & PZ (1)	0.0012059 (0.0009726)	0.0005164 (0.0009769)	0.0012197 (0.0009895)
FF (1/3)	−0.0006977 (0.0041535)	−0.0011386 (0.0041541)	−0.0001076 (0.0041603)
FF (2/3)	−0.0044559* (0.0024214)	0.0029826 (0.0024271)	0.0039919* (0.0024382)
FF (1)	0. 0,029,672* (0.0016783)	0.001853 (0.001684)	0.0029658* (0.0017014)

*Note:* Regressors in the base model (BM) regression model include student age, race, gender, rural/urban area of residence, participation in school free/reduced lunch program, year dummy variables, and lag of the included restaurant count in the model. BM + grade FE has all the variables in BM plus the grade fixed effects. The last column reports estimates from a model that has all variables as in BM, grade fixed effects and the commercial food environment around student residence. School-level clustered standard errors are in parentheses. N = 111,464.

*** Significant at the 1 percent level.

** Significant at the 5 percent level.

* Significant at the 10 percent level.

**Table A3:** Estimates of density of fastfood restaurants (ff), sandwich places (SW) and pizzerias (PZ) surrounding schools on student’s BMI z-score among **HIGH** school students.

Variable (miles)	Base Model (BM)	BM + grade FE	BM + grade FE + Food Environment
FF, SW & PZ (1/3)	−0.0005556 (0.0017172)	−0.0020203 (0.0017252)	−0.000279 (0.0017389)
FF, SW & PZ (2/3)	−0.0014892 (0.0009326)	−0.0023548*** (0.0009371)	−0.0013104 (0.0009524)
FF, SW & PZ (1)	−0.0015765*** (0.0006368)	−0.002141*** (0.0006427)	−0.0014124** (0.0006548)
FF (1/3)	−0.0039097 (0.0024319)	−0. 0,063,118*** (0.0024509)	−0.0044763* (0.002463)
FF (2/3)	−0.0041524*** (0.0016249)	−0.0057146*** (0.001637)	−0.0042166** (0.0016572)
FF (1)	−0.0042261*** (0.0011196)	−0.0054536*** (0.0011324)	−0.0043714*** (0.0011503)

*Note:* Regressors in the base model (BM) regression model include student age, race, gender, rural/urban area of residence, participation in school free/reduced lunch program, year dummy variables, and lag of the included restaurant count in the model. BM + grade FE has all the variables in BM plus the grade fixed effects. The last column reports estimates from a model that has all variables as in BM, grade fixed effects and the commercial food environment around student residence. School-level clustered standard errors are in parentheses. N = 221,715.

*** Significant at the 1 percent level.

** Significant at the 5 percent level.

* Significant at the 10 percent level.

**Table A4:** Estimates (OLS) of density of fastfood restaurants (FF), sandwich places (SW) and pizzerias (PZ) surrounding schools on student’s BMI z-score among **RURAL** students.

Variable (miles)	Base Model (BM)	BM + grade FE	BM + grade FE + Food Environment
FF, SW & PZ (1/3)	0.0034052 (0.0030909)	0.0021895 (0.0030897)	0.0027738 (0.0030954)
FF, SW & PZ (2/3)	0.000234 (0.0015692)	−0.0000079 (0.0015685)	0.0006429 (0.0015744)
FF, SW & PZ (1)	0.0000137 (0.0010542)	−0.0002711 (0.0010552)	0.0002876 (0.0010611)
FF (1/3)	−0.000744 (0.0046296)	−0.0026651 (0.0046301)	0.0025971 (0.0046365)
FF (2/3)	−0.000939 (0.0025067)	−0.0014221 (0.0025068)	−0.000964 (0.0025119)
FF (1)	−0.000807 (0.0016724)	−0.0015569 (0.001675)	−0.0009451 (0.0016808)

*Note:* Regressors in the base model (BM) regression model include student age, race, gender, rural/urban area of residence, participation in school free/reduced lunch program, year dummy variables, and lag of the included restaurant count in the model. BM + grade FE has all the variables in BM plus the grade fixed effects. The last column reports estimates from a model that has all variables as in BM, grade fixed effects and the commercial food environment around student residence. School-level clustered standard errors are in parentheses. N = 216,181.

* Significant at the 10 percent level.

** Significant at the 5 percent level.

*** Significant at the 1 percent level.

**Table A5:** Estimates (OLS) of density of fastfood restaurants (FF), sandwich places (SW) and pizzerias (PZ) surrounding schools on student’s BMI z-score among **URBAN** students.

Variable (miles)	Base Model (BM)	BM + grade FE	BM + grade FE + Food Environment
FF, SW & PZ (1/3)	0.00,072 (0.0013826)	−0.000646 (0.0013905)	−0.0005268 (0.0013925)
FF, SW & PZ (2/3)	0.0001783 (0.0006466)	−0.0003812 (0.0006506)	−0.000294 (0.0006544)
FF, SW & PZ (1)	−0.0001177 (0.000418)	−0.0004601 (0.0004213)	−0.0002951 (0.0004253)
FF (1/3)	0.00215 (0.0019744)	−0.0002487 (0.0019886)	−0.0002821 (0.0019905)
FF (2/3)	−0.0005211 (0.0010765)	−0.0013552 (0.0010837)	−0.0012909 (0.0010883)
FF (1)	−0.001178 (0.0007297)	−0.0018809** (0.0007357)	−0.0016462** (0.0007409)

*Note:* Regressors in the base model (BM) regression model include student age, race, gender, rural/urban area of residence, participation in school free/reduced lunch program, year dummy variables, and lag of the included restaurant count in the model. BM + grade FE has all the variables in BM plus the grade fixed effects. The last column reports estimates from a model that has all variables as in BM, grade fixed effects and the commercial food environment around student residence. School-level clustered standard errors are in parentheses. N = 392,736.

* Significant at the 10 percent level.

** Significant at the 5 percent level.

*** Significant at the 1 percent level.

## Abbreviations:

ACHI: arkansas center for health improvement
BMI: body mass index
CDC: centers for disease control and prevention
GIS: geographic information system
IV: instrumental variable(s)
